# Enthorinal phase precession revisited – single-run analysis of *in-vivo* grid cell data

**DOI:** 10.1186/1471-2202-12-S1-P288

**Published:** 2011-07-18

**Authors:** Eric T Reifenstein, Martin B Stemmler, Andreas VM Herz, Susanne Schreiber

**Affiliations:** 1 Institute for Theoretical Biology, Humboldt-Universität zu Berlin, Berlin, Germany; 2 Department Biology II, Ludwig-Maximilians-Universität, Planegg-Martinsried, Germany

## 

When a rat explores its environment, grid cells in the medial entorhinal cortex show increased activity at specific locations that constitute a regular hexagonal grid. As the rat enters and progresses through one of these “grid fields” on a linear track, spikes occur at successively earlier phases in the LFP's theta rhythm. This phenomenon is called phase precession. For rats foraging in two-dimensional environments, however, phase precession has not yet been quantified. Unlike on the linear track, a rat does not repeat the same path over and over again in an open arena; pooling different runs to assess phase precession becomes fraught with difficulty. Instead, we analyze grid cell spike trains recorded by [1] on a run-by-run basis, and do the same also for linear track data (by [2]). Surprisingly, even on the linear track, phase precession during single runs is stronger than the average phase precession in the pooled data. We show that a grid cell spike’s theta phase allows one to estimate the animal’s position on a linear track to within about 10 % of the size of a typical grid field. The spectrum of grid cell spike trains recorded in a two-dimensional environment reveals a peak that is shifted by approximately 1 Hz relative to the peak in the LFP theta rhythm, indicative of robust phase precession. We investigate the dependence of spike phase on the radial distance from the grid field center and how phase precession depends on whether the path is transverse or tangential to the grid field, in order to create a model for decoding position in two dimensions from spike phases.
